# Breast cancer survival and mortality among women with type 2 diabetes: a retrospective cohort study

**DOI:** 10.1038/s41598-025-08785-7

**Published:** 2025-07-18

**Authors:** Alison J. Sears, Sarah H. Wild, Ines Mesa-Eguiagaray, Peter S. Hall, Jonine D. Figueroa

**Affiliations:** 1 Usher Institute, University of Edinburgh, Edinburgh, UK; 2 Institute of Genetics and Cancer, University of Edinburgh, Edinburgh, UK; 3 Division of Cancer Epidemiology and Genetics, National Cancer Institute, Bethesda, MD USA

**Keywords:** Breast cancer, Type 2 diabetes, Molecular subtype, Survival, Cox proportional hazard models, Breast cancer, Epidemiology, Type 2 diabetes

## Abstract

Type 2 diabetes (T2DM) is estimated to affect over 200 million women globally and over 2 million women are estimated to be diagnosed with breast cancer each year. This study aimed to determine the association between breast cancer molecular subtypes and survival in a cohort of women with T2DM. A retrospective cohort study was conducted using linked Scottish population-based data for 3,042 women diagnosed with T2DM prior to their diagnosis of invasive breast cancer between 2010 and 2019 that had complete data available. The women in the cohort were aged 50 to 84 years at the time of breast cancer diagnosis. Univariate analyses were performed using non-parametric ten-year Kaplan Meier (KM) estimates for breast cancer survival by molecular subtype. Hazard ratios (HR) for breast cancer mortality within three years were estimated using Cox proportional hazard models, adjusting for age at breast cancer diagnosis, year of diagnosis, Scottish region, mode of detection, treatment, area-based socioeconomic status, and T2DM duration were conducted. Multiple imputation was performed as a sensitivity analysis. The distribution of molecular subtype based on St Gallen’s criteria was luminal A (58%), luminal B (HER2-) (19.2%), triple negative (9.9%), luminal B (HER2 +) (8.5%), and HER2-enriched (4.4%). There were 286 breast cancer deaths in total within three years. KM estimates showed women with HER2-enriched tumours had the worst survival at ages 50 to 69, while those with triple-negative tumours had the worst survival at ages 70 to 84. Adjusted HRs (95% confidence intervals) for mortality over 3 years compared to luminal A were 2.04 (1.43, 2.90) for luminal B (HER2-), 5.68 (3.40, 9.50) for triple negative, 2.22 (1.46, 3.38) for luminal B (HER2 +), and 2.93 (1.63, 5.27) for HER2-enriched. The imputed cohort adjusted HRs were attenuated, particularly for HER2-enriched (HR = 1.71 (1.01, 2.89)) and triple negative (HR = 2.74 (1.72, 4.37)). Like the general population, triple negative and HER2-enriched tumors had worse prognosis compared to luminal A tumors and their association with breast cancer mortality are similar in women with type 2 diabetes and the general population. However, our findings revealed differences in the survival ranking of HER2-enriched and triple-negative subtypes for breast cancer diagnosed in the 50–69 age group between women with type 2 diabetes and the general population, warranting further exploration.

## Introduction

The rising prevalence of diabetes presents a significant global health challenge, with projections indicating that by 2030, approximately 10.2% of the world’s population will be living with the disease^1^. Type 2 diabetes mellitus (T2DM) accounts for over 95% of all diabetes cases and is associated with a range of complications and co-morbidities^2^ and has been identified as a risk factor for breast cancer^3,4^. Women with pre-existing diabetes may be at a higher risk for late-stage tumours, larger tumour size, and lymph node invasiveness compared to those without a prior diabetes diagnosis^5^. However, the factors that could affect survival among women with T2DM and breast cancer are complex and not fully understood, given the varying quality of evidence and study limitations^5^. In vitro studies have shown hyperglycaemia and hyperinsulinemia can increase breast cancer cell proliferation, invasiveness, and migration^6^. Moreover, a study found high levels of glucose enhanced migration in oestrogen receptor-positive breast cancer cells, suggesting that the hormone receptor pathways may be involved in mediating the response of breast cancer cells to metabolic changes in the microenvironment^7^.

Breast cancer is the most common malignancy among women globally, and the incidence is increasing^8,9^. In Scotland, one in eight women is diagnosed with breast cancer during their lifetime^10^. Breast cancer can be classified into one of four intrinsic molecular subtypes which are based on gene expression, and each contains distinct molecular characteristics, providing information on prognosis and the optimal course of treatment^11,12^. The St. Gallen Expert Panel recommends classifying the intrinsic molecular subtypes using immunohistochemistry (IHC) markers as surrogates: oestrogen receptor (ER), progesterone receptor (PR), human epidermal growth factor receptor 2 (HER2), and the proliferation marker Ki-67 or tumour grade^13^. Based on St. Gallen’s, five molecular subtypes can be defined: luminal A-like, luminal B-like (HER2 −), luminal B-like (HER2 +), HER2-enriched, and triple-negative breast cancer (TNBC)^13^. Luminal-like cancers are the most common in European ancestry populations indicating responsiveness to hormone therapy^14^. TNBC is an aggressive subtype shown in many to have poor prognosis with limited targeted therapies^15^.

Scotland has been collecting high-quality electronic health records for several decades. The Scottish Care Information (SCI)-Diabetes dataset has had nationwide coverage since 2006. The dataset is estimated to capture over 99% of people with a diabetes diagnosis in Scotland^16^. SCI-diabetes is updated daily, and all general practices and hospital clinics in the country contribute data to comprise a cohort with over 4 million person-years of follow-up^16^. In 2020, 88.1% of individuals had a T2DM diagnosis^16^. The Scottish Cancer Registry (SMR06) is a national electronic database that has existed since 1981 with breast cancer completeness of 99%^17,18^. ER data collection began in 1997, and PR and HER2 data collection began in 2009^18^. SMR06 contains data on breast cancer detection mode sourced from Scotland’s national mammography programme. Women aged 50 to 69 are invited for mammography every three years^19^. Another key prognostic factor included in SMR06 is the Scottish Index of Multiple Deprivation (SIMD) https://www.gov.scot/collections/scottish-index-of-multiple-deprivation-2020/, a nationwide area-based measurement of socioeconomic status (SES). SIMD was established by the Scottish Government and is based on measures of income, employment, health, education, crime, access to services, and housing. People registered with a Scottish general practitioner are assigned a unique Community Health Index (CHI) number which is an identifier on all public health system records in the country that allows data linkage. Pseudonymised extracts of SCI-diabetes are linked to SMR06 in the Scottish Diabetes Research Network (SDRN) national dataset (NDS) ^16^.

This project aimed to investigate the relationship between distinct molecular subtypes and survival among women with T2DM and breast cancer in Scotland using a retrospective cohort study design.

## Methods

### Study cohort

The cohort consisted of women diagnosed with T2DM before diagnosis of a primary invasive breast cancer (International Classification of Diseases (ICD), 10th revision, code C50). T2DM diagnosis was obtained from linked primary and secondary care records^16^.

All women with T2DM aged 50 to 84 years of age with breast cancer diagnoses between 2010 and 2019 were identified. Since the SMR06 began recording PR and HER2 in 2009, only women diagnosed after 2010 were included allowing one year for the collection of these additional variables. The 2019 cut-off was established to avoid the effects of the Covid-19 pandemic which caused delays in diagnoses and accurate data collection. The cohort age restriction (50 to 84 years) was chosen to include women who are of breast cancer screening age (50 to 69 years) and to minimize the amount of missing data, which was more common in women over the age of 84 years (data not shown).

**Fig. 1:**
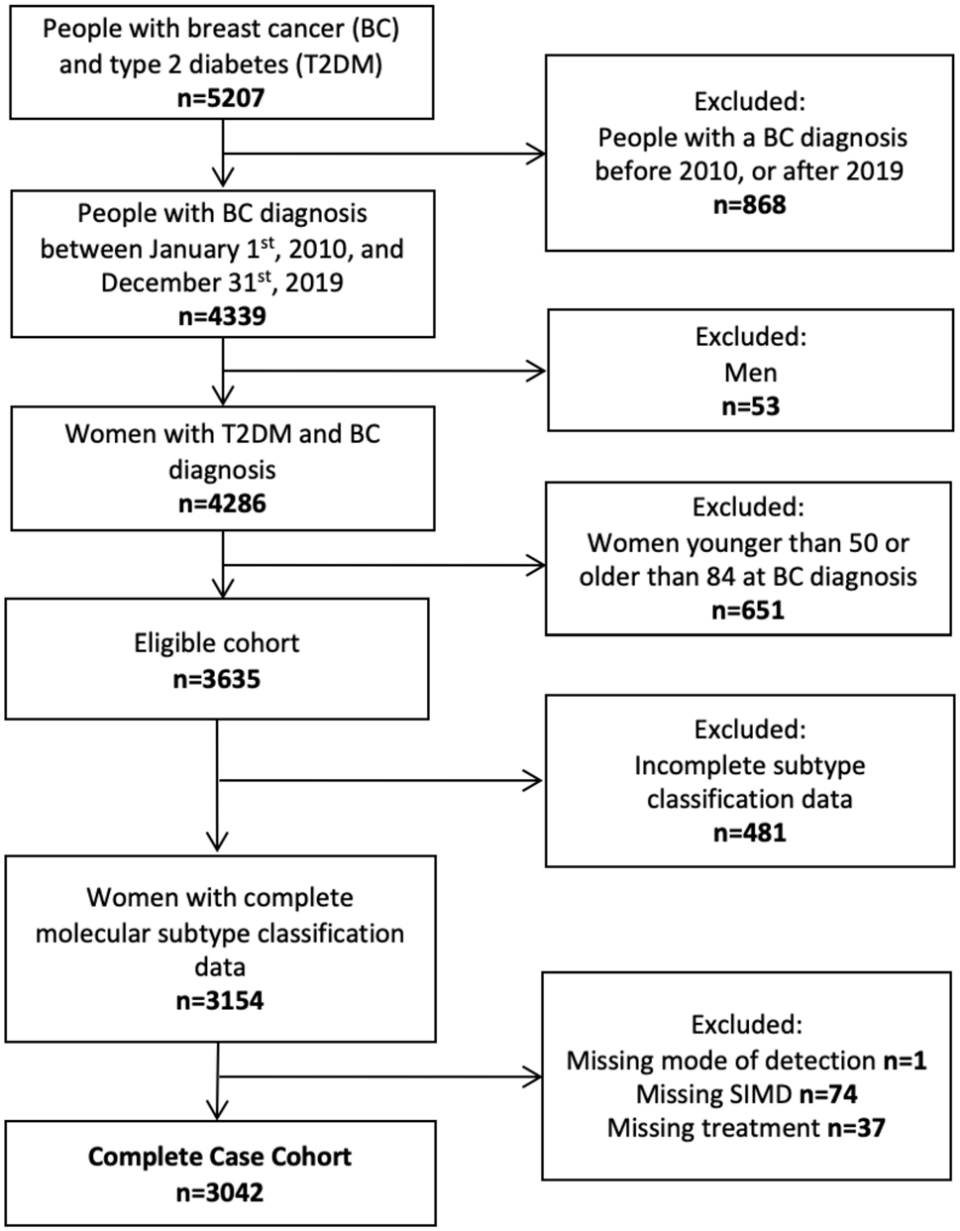
Flowchart of the complete case cohort. The cohort consisted of women with T2DM aged 50 to 84 years with breast cancer diagnosis between 2010 and 2019 in Scotland.

Women with other primary malignant cancers prior to breast cancer diagnosis were excluded from the analysis. Individuals were also removed if they had a matching date of breast cancer incidence and date of death, or a breast cancer diagnosis only from their death certificate. Moreover, women with missing data for the following variables were excluded: age, T2DM duration prior to breast cancer diagnosis, death censoring and cause, Scottish region, breast cancer diagnosis year, ER and/or PR status, HER2 status, grade (only for women with luminal cancers), mode of detection, Scottish Index of Multiple Deprivation (SIMD), treatment information (surgery, chemotherapy, radiotherapy, and hormone therapy) (Fig. 1).

### Definitions

#### Breast cancer

Breast cancer receptor status was assigned through IHC staining and molecular subtypes were classified based on St. Gallen criteria, utilizing grade (I—well-differentiated to III—poorly differentiated) as a proxy for Ki-67 in luminal tumours because Ki-67 is not used routinely in Scotland^13^. The surrogate markers ER + and/or PR + /HER2- (grade I/II) characterized Luminal A tumours, ER + and/or PR + /HER2- (grade III) for Luminal B (HER2-) tumours, ER + and/or PR + /HER2 + for Luminal B (HER2 +) tumours, ER-/PR-/HER2 + for HER2-enriched tumours, and ER-/PR-/HER2- for TNBC tumours.

#### Type 2 diabetes mellitus

T2DM status was assigned based on cleaned data regarding diabetes type in the research dataset. T2DM duration at breast cancer diagnosis was calculated as the time difference between the date of the earliest mention of T2DM in the SCI-diabetes research dataset and the diagnosis date of breast cancer in SMR06^16^.

#### Mode of detection

The mode of detection was categorized into screen-detected or non-screen-detected^19^.

#### Deprivation

For this analysis, the 2016 version was used and SIMD was expressed in quintiles ranging from the most deprived (quintile 1) to the least deprived (quintile 5)^20^.

#### Scottish region

Regions were categorized based on the individual’s residence at the time of breast cancer diagnosis, using Scotland’s 14 National Health Service (NHS) boards which were grouped into three regions (West, North, and South-east) for analysis. The West region comprised Ayrshire and Arran, Forth Valley, Clyde and Lanarkshire, and Greater Glasgow. The North comprised the Western Isles, Grampian, Highland, Orkney, Shetland, and Tayside. South-east comprised Borders, Dumfries and Galloway, Fife, and Lothian^21^.

### Survival

Breast cancer deaths were identified from the primary cause of death in death records. The complete follow-up duration was defined as the time from breast cancer diagnosis (recorded in SMR06 as the first consultation or hospital admission for breast cancer) to the time of death, or until November 30th, 2021^22^. Follow-up was censored at the date of other causes of death Non-parametric Kaplan–Meier estimates were used to describe breast cancer survival over a ten- year period by molecular subtype for two age categories: breast cancer screening-eligible age (50 to 69 years) and not routinely eligible for screening age (70 to 84 years).

Cox proportional hazard models were fitted for univariate and multivariable analyses to assess breast cancer survival and various prognostic factors over a maximum of three years of follow-up to maximise the size of the dataset and reduce proportional hazard violations that occurred at five years. Models were adjusted for the following covariates: molecular subtype, age at breast cancer diagnosis, year of breast cancer diagnosis, Scottish region, mode of detection, treatment (binary variables for surgery, chemotherapy, radiotherapy, and hormone therapy), SIMD quintile, and T2DM duration. To assess the proportionality, Schoenfeld’s tests were conducted for each variable. All analyses were carried out using R 4.6^23^.

Multiple imputation was performed as sensitivity analysis and compared to the results from complete case models. Missing covariate data for ER status, PR status, HER2 status, grade, SIMD, mode of detection and treatments were imputed by chained equation models using a model compatible with the analysis model^24^. The outcome used was the Nelson-Aalen estimator for time to death and a censoring indicator as described by White and Royston^25^. Of the initial 3635 cases, 16% had a missing value for at least one variable so 30 imputations were used to give reliable estimates^24^. Each imputed dataset was analysed and the Rubin’s rule was used to combine the coefficients from the models^26^.

## Results

### Characteristics of cohort by vital status

Between 2010 and 2019, 3635 women between the ages of 50 and 84 with T2DM were diagnosed with breast cancer in Scotland (Fig. 1). Of these women, 3042 (84%) had no missing data (Fig. 1, Table 1). Over half of the women had luminal A cancer (58%), followed by luminal B (HER2-, 19.2%), TNBC (9.9%), luminal B (HER2 + , 8.5%), and HER2-enriched (4.4%). The group with HER2-enriched tumours had the highest proportion of breast cancer deaths (34.1%) over the follow-up period (median follow-up [IQR] = 4.59 years [2.77, 7.28]) and the group with luminal A tumours had the lowest proportion of breast cancer deaths during this period (8.3%) (Table 1). Higher proportions of women 70 to 84 years of age had breast cancer deaths (17.7%) compared to younger women (10.2%). A third of women (33.7%) had a screen-detected breast cancer and of those women, only 5.4% had breast cancer deaths, whereas women with non-screen detected diagnoses had a much higher proportion of breast cancer deaths (18.1%). Among treatment categories, women who received chemotherapy had the highest proportion of breast cancer deaths (20.1%), followed by radiotherapy (11.0%), hormone therapy (11.0%), and surgery (8.5%). There was no distinct pattern in the proportions of breast cancer deaths and deprivation, Scottish region, or T2DM duration. In contrast, differences in proportions of breast cancer deaths were observed between different molecular subtypes, age groups, year at breast cancer diagnosis, mode of detection, and treatments (Table 1).

**Table 1:** Descriptive characteristics of women with complete covariate data diagnosed with breast cancer and type 2 diabetes.

Characteristics	Total	Alive		BC death		Other death	
	n = 3042	n = 1964 [64.6%]		n = 421 [13.8%]		n = 657 [21.6%]	
	n	n	%	n	%	n	%
Subtype							
Luminal A	1764	1229	[69.7]	147	[8.3]	388	[22.0]
Luminal B (HER2-)	583	359	[61.6]	89	[15.2]	135	[23.2]
Luminal B (HER2 +)	259	156	[60.2]	48	[18.5]	55	[21.3]
HER2-enriched	135	60	[44.4]	46	[34.1]	29	[21.5]
TNBC	301	160	[53.2]	91	[30.2]	50	[16.6]
Age at BC diagnosis							
50–69 years	1558	1217	[78.1]	158	[10.2]	183	[11.7]
70–84 years	1484	747	[50.3]	263	[17.7]	474	[32.0]
Year of BC diagnosis							
2010–2013	1065	539	[50.6]	183	[17.2]	343	[32.2]
2014–2016	898	597	[66.5]	124	[13.8]	177	[19.7]
2017–2019	1079	828	[76.7]	114	[10.6]	137	[12.7]
Mode of detection							
Screen-detected	1027	848	[82.6]	56	[5.4]	123	[12.0]
Non-screen-detected	2015	1116	[55.4]	365	[18.1]	534	[26.5]
Surgery							
Yes	2524	1845	[73.1]	215	[8.5]	464	[18.4]
No	518	119	[23.0]	206	[51.6]	193	[48.4]
Chemotherapy							
Yes	612	424	[69.3]	123	[20.1]	65	[10.6]
No	2430	1540	[63.4]	298	[12.3]	592	[24.3]
Radiotherapy							
Yes	1832	1352	[73.8]	201	[11.0]	279	[15.2]
No	1210	612	[50.6]	220	[18.2]	378	[31.2]
Hormone therapy							
Yes	2449	1632	[66.6]	270	[11.0]	547	[22.4]
No	593	332	[56.0]	151	[25.5]	110	[18.5]
SIMD							
5 (least deprived)	408	283	[69.4]	62	[15.2]	63	[15.4]
4	586	405	[69.1]	64	[10.9]	117	[20.0]
3	567	369	[65.1]	77	[13.6]	121	[21.3]
2	732	449	[61.3]	111	[15.2]	172	[23.5]
1 (most deprived)	749	458	[61.1]	107	[14.3]	184	[24.6]
Scottish region							
West	1417	944	[66.6]	196	[13.8]	277	[19.6]
North	722	468	[64.8]	94	[13.0]	160	[22.2]
Southeast	903	552	[61.1]	131	[14.5]	220	[24.4]
T2DM duration							
< 5 years	977	675	[69.1]	129	[13.2]	173	[17.7]
5–9 years	766	514	[67.1]	104	[13.6]	148	[19.3]
10–14 years	547	345	[63.1]	73	[13.3]	129	[23.6]
15–19 years	284	156	[54.9]	48	[16.9]	80	[28.2]
≥ 20 years	468	274	[58.5]	67	[14.3]	127	[27.2]

Women in the cohort had a breast cancer diagnosis in Scotland between 2010 and 2019 and were stratified by vital status at the end of follow-up in November 2021 and cause of death.

Brackets [] indicate row percentages.

*BC* breast cancer, *TNBC* triple negative breast cancer, *HER2* human epidermal growth factor 2, *T2DM* type 2 diabetes mellitus, *SIMD* Scottish index of multiple deprivation.

Among the eligible cohort of 3635 women, proportions of missingness in variables were highest for PR status (17.99%), tumour grade (11.17%), and HER2 status (6.82%). All other variables had less than 2.5% missingness (Table 2). The distribution of descriptive characteristics was similar between the complete case and imputed cohorts (Table 3) with a slightly higher proportion of luminal A tumours and lower proportion of other tumours in the imputed cohort.

**Table 2:** Missingness table of eligible cohort.

Characteristics	Missingness of eligible cohort	
	n = 3635	
	n	%
PR status	654	[17.99]
Grade	406	[11.17]
HER2 status	248	[6.82]
ER status	88	[2.42]
SIMD quintile	86	[2.37]
Hormone therapy	56	[1.54]
Radiotherapy	11	[0.30]
Chemotherapy	8	[0.22]
Mode of detection	5	[0.14]
Surgery	3	[0.08]

The eligible cohort consisted of women diagnosed with breast cancer in Scotland between 2010 and 2019, aged 50–84 years at diagnosis.

Complete characteristics: vital status, age at BC diagnosis, year of BC diagnosis, Scottish region, T2DM duration.

Brackets [] indicate row percentages.

*BC* breast cancer, *ER* oestrogen receptor, *PR* progesterone receptor, *HER2* human epidermal growth factor 2, *T2DM* type 2 diabetes mellitus, *SIMD* Scottish index of multiple deprivation.

**Table 3:** Descriptive characteristics of eligible (complete case) and imputed cohort.

Characteristics	Eligible cohort		Imputed cohort	
	n	%	n	%
Subtype				
Luminal A	1489	[56.4]	2137	[58.8]
Luminal B (HER2-)	522	[19.8]	705	[19.4]
Luminal B (HER2 +)	230	[8.7]	289	[8.0]
HER2-enriched	116	[4.4]	154	[4.2]
TNBC	281	[10.7]	350	[9.6]
ER status				
Negative	498	[14.0]	536	[14.8]
Positive	3049	[86.0]	3099	[85.2]
PR status				
Negative	807	[27.1]	994	[27.3]
Positive	2174	[72.9]	2641	[72.7]
HER2 status				
Negative	2981	[88.0]	3192	[87.8]
Positive	406	[12.0]	443	[12.2]
Grade				
1	393	[12.2]	440	[12.1]
2	1705	[52.8]	1919	[52.8]
3	1131	[35.0]	1276	[35.1]
Mode of detection				
Screen-detected	1146	[31.6]	1146	[31.5]
Non-screen-detected	2484	[68.4]	2489	[68.5]
Surgery				
Yes	2747	[75.6]	2749	[75.6]
No	885	[24.4]	886	[24.4]
Chemotherapy				
Yes	669	[18.4]	670	[18.5]
No	2958	[81.6]	2964	[81.5]
Radiotherapy				
Yes	1971	[54.4]	1978	[54.4]
No	1653	[45.6]	1657	[45.6]
Hormone therapy				
Yes	2846	[79.5]	2893	[79.6]
No	733	[20.5]	742	[20.4]
SIMD				
5 (least deprived)	476	[13.4]	491	[13.5]
4	675	[19.0]	695	[19.1]
3	655	[18.5]	672	[18.5]
2	849	[23.9]	868	[23.9]
1 (most deprived)	894	[25.2]	910	[25.0]

The eligible cohort consisted of women diagnosed with breast cancer in Scotland between 2010 and 2019, aged 50–84 years at diagnosis. The imputed cohort refers to the eligible cohort with missing variables imputed.

Complete characteristics: age at BC diagnosis, year of BC diagnosis, Scottish region, T2DM duration.

Brackets [] indicate row percentages.

*BC* breast cancer, *ER* oestrogen receptor, *PR* progesterone receptor, *HER2* human epidermal growth factor 2,* TNBC* triple negative breast cancer*T2DM* type 2 diabetes mellitus, *SIMD* Scottish index of multiple deprivation.

### Survival

**Fig. 2:**
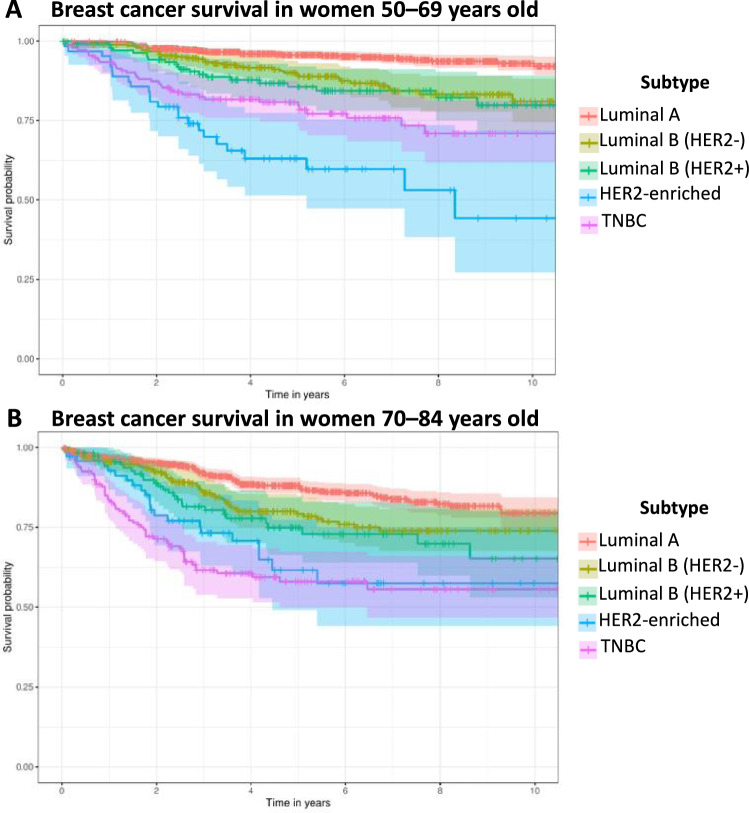
Kaplan–Meier curves demonstrating breast cancer specific survival by molecular subtype. Breast cancer specific survival of women with type 2 diabetes and diagnosed with breast cancer in Scotland from 2010 to 2019, aged 50 to 69 (**A**) and 70 to 84 (**B**) (95% confidence intervals are represented by shading).

Women with luminal A breast cancer had the best breast cancer survival over ten years in both age categories (Fig. 2). Women with HER2-enriched tumours had the worst survival for those diagnosed between the ages of 50 to 69, whereas women with TNBC tumours had the worst survival for those diagnosed between the ages of 70 and 84 (Fig. 2, Table S1).

In both the minimally adjusted and adjusted complete case Cox models that are described in Table 4, breast cancer mortality over a 3-year follow-up period was higher among women with other tumour types compared to those with luminal A tumours. Among the other molecular subtypes, the lowest excess mortality occurred among women with luminal B (HER2-) tumours, followed by those with luminal B (HER2 +) tumours, HER2-enriched tumours, and TNBC tumours.

**Table 4:** Minimally adjusted and adjusted Cox models of breast cancer mortality in complete case and imputed cohort.

	Minimally adjusted complete case (95% CI)	Adjusted complete case (95% CI)	Minimally adjusted imputed (95% CI)	Adjusted imputed (95% CI)
Subtypes				
Luminal A	Ref	Ref	Ref	Ref
Luminal B (HER2-)	1.85 (1.31, 2.61)	2.04 (1.43, 2.90)	1.81 (1.36, 2.42)	1.92 (1.40, 2.62)
Luminal B (HER2 +)	2.83 (1.89, 4.23)	2.22 (1.46, 3.38)	2.38 (1.66, 3.42)	2.16 (1.46, 3.18)
HER2-enriched	5.82 (3.91, 8.66)	2.93 (1.63, 5.27)	4.68 (3.26, 6.72)	1.71 (1.01, 2.89)
TNBC	6.28 (4.63, 8.53)	5.68 (3.40, 9.50)	4.70 (3.58, 6.17)	2.74 (1.72, 4.37)
Age at BC diagnosis				
50–69 years	Ref	Ref	Ref	Ref
70–84 years	2.19 (1.72, 2.79)	1.19 (0.88, 1.60)	2.08 (1.69, 2.55)	1.17 (0.91, 1.50)
Year of BC diagnosis				
2010–2013	Ref	Ref	Ref	Ref
2014–2016	0.85 (0.64, 1.14)	0.81 (0.60, 1.08)	0.82 (0.65, 1.04)	0.81 (0.64, 1.03)
2017–2019	0.94 (0.72, 1.24)	0.78 (0.58, 1.03)	0.84 (0.65, 1.05)	0.81 (0.63, 1.02)
Mode of detection				
Screen-detected	Ref	Ref	Ref	Ref
Non-screen-detected	5.24 (3.42, 8.02)	2.52 (1.62, 3.92)	4.83 (3.38, 6.89)	1.80 (1.24, 2.62)
Surgery				
Yes	Ref	Ref	Ref	Ref
No	11.37 (8.74, 14.78)	14.02 (10.52, 18.69)	9.86 (7.89, 12.33)	14.06 (10.83, 18.24)
Chemotherapy				
Yes	Ref	Ref	Ref	Ref
No	0.40 (0.31, 0.53)	0.71 (0.51, 0.97)	0.45 (0.35, 0.56)	0.75 (0.57, 0.99)
Radiotherapy				
Yes	Ref	Ref	Ref	Ref
No	1.83 (1.44, 2.32)	0.71 (0.53, 0.93)	2.25 (1.82, 2.77)	0.83 (0.65, 1.06)
Hormone therapy				
Yes	Ref	Ref	Ref	Ref
No	3.62 (2.86, 4.58)	4.15 (3.16, 5.44)	3.57 (2.93, 4.35)	3.20 (2.19, 4.69)
SIMD quintile				
5 (least deprived)	Ref	Ref	Ref	Ref
4	0.74 (0.49, 1.12)	0.79 (0.52, 1.20)	0.84 (0.67, 1.06)	0.94 (0.75, 1.19)
3	0.81 (0.54, 1.22)	0.93 (0.61, 1.40)	0.88 (0.70, 1.12)	0.99 (0.77, 1.26)
2	1.00 (0.69, 1.46)	0.90 (0.61, 1.31)	1.11 (0.89, 1.38)	0.98 (0.78, 1.24)
1 (most deprived)	1.04 (0.71, 1.53)	0.85 (0.58, 1.26)	1.25 (0.99, 1.59)	1.15 (0.90, 1.46)
Scottish region				
West	Ref	Ref	Ref	Ref
North	0.88 (0.65, 1.19)	0.77 (0.56, 1.06)	0.77 (0.60, 1.00)	0.86 (0.66, 1.12)
Southeast	1.11 (0.85, 1.44)	0.91 (0.69, 1.21)	0.93 (0.74, 1.17)	1.12 (0.88, 1.42)
T2DM duration				
< 5	Ref	Ref	Ref	Ref
5–9	1.04 (0.76, 1.44)	0.99 (0.72, 1.36)	0.96 (0.73, 1.25)	1.01 (0.77, 1.32)
10–14	0.92 (0.64, 1.33)	0.79 (0.55, 1.15)	0.88 (0.65, 1.18)	0.78 (0.57, 1.06)
15–19	1.54 (1.05, 2.27)	1.14 (0.77, 1.68)	1.34 (0.97, 1.87)	1.15 (0.82, 1.61)
≥ 20	1.09 (0.76, 1.57)	0.91 (0.63, 1.33)	0.99 (0.74, 1.34)	0.89 (0.66, 1.22)

Cox models indicate breast cancer mortality (with 95% CI) up to three years after breast cancer diagnosis**,** among women with type 2 diabetes and a breast cancer diagnosis in Scotland between 2010 and 2019.

Minimally adjusted models: age at breast cancer diagnosis, year of diagnosis, Scottish region.

Adjusted models: age at breast cancer diagnosis, year of diagnosis, Scottish region, mode of detection, treatments (surgery, chemotherapy, radiotherapy, hormone therapy), SIMD quintile and T2DM duration.

*BC* breast cancer, *TNBC* triple negative breast cancer, *HER2* human epidermal growth factor 2, *CI* confidence interval, *T2DM* type 2 diabetes mellitus, *SIMD* Scottish index of multiple deprivation, *Ref* reference category.

In the adjusted model, breast cancer mortality was higher for women who had non-screen-detected tumours compared to those who had screen-detected tumours (HR = 2.52, 95% CI: 1.62 to 3.92, Table 4), for women who did not undergo surgery compared to those who did undergo surgery (HR = 14.02, 95% CI: 10.52 to 18.69), and for women who did not have hormone therapy compared to those that did have hormone therapy (HR = 4.15, 95% CI: 3.16 to 5.44). Breast cancer mortality was notably lower among those who did not receive chemotherapy compared to those who did receive chemotherapy (HR = 0.71, 95% CI: 0.51 to 0.97), and women who did not undergo radiotherapy compared to those who did undergo radiotherapy (HR = 0.71, 95% CI: 0.53 to 0.93). The adjusted model indicated no associations between breast cancer mortality and age at breast cancer diagnosis, year of breast cancer diagnosis, SIMD quintiles, Scottish region, or TD2M duration. Notably, the strongest predictors of survival were the mode of detection and treatments (Table 4).

Compared to the complete case cohort, the imputed cohort showed lower HRs for breast cancer specific mortality across molecular subtypes in both the minimally adjusted and adjusted models (Table 4). The minimally adjusted model followed the same pattern of excess mortality by molecular subtype as the complete case cohort, however, the fully adjusted model had attenuated HRs for all subtypes when compared to the estimates from the complete case model. The difference in HRs was greatest amongst HER2-enriched tumours (HR was 1.71 for imputed cohort compared to 4.68 in the complete case cohort) and TNBC (HR was 2.74 in the imputed cohort compared to 4.70 in the complete case cohort) subtypes.

## Discussion

As expected, among women with T2DM, mortality was lowest for those diagnosed with luminal A tumours. These findings are consistent with prior research from the general population with breast cancer in Scotland^27^. In the general Scottish breast cancer population aged 50 to 69, the lowest crude 5-year breast cancer specific survival (95% CI), regardless of diabetes status, was observed among women with TNBC tumours at 78.6 compared to 81.7 for HER2-enriched tumours and 95.5% for luminal A tumours^27^. In contrast, among women with T2DM and breast cancer diagnosed between the ages of 50 and 69 years, the lowest crude 3-year breast cancer specific survival was among those with HER2-enriched tumours was about 8% lower compared to 78.2 for TNBC tumours and 93.8 for luminal A tumours. Results from our adjusted Cox models after imputation for women with T2DM and breast cancer death relative to luminal A tumours showed HER2-enriched tumours almost 2X more likely to die and 3X more likely to die if they had TNBC tumours. Our results show the importance of performing sensitivity analysis and imputation of tumour characteristics in special populations such as women with diabetes.

Due to the aggressive nature of HER2-enriched tumours, improvement in prognosis is reliant on chemotherapy^28^. However, patients with diabetes often experience heightened chemotoxicity levels, potentially influencing survival outcomes^29^. Moreover, research has shown that diabetes has a negative effect in postoperative cases of HER2-positive breast cancer patients treated with trastuzumab^30^. While we observe higher mortality for HER2-enriched tumours in complete case analysis these estimates were attenuated upon imputation for tumour characteristics suggesting current treatment may be sufficient, however, there is some suggestion that other treatment approaches may be warranted to improve outcomes in women with diabetes and breast cancer that should be pursued in future research^30^.

Adjusted Cox models indicated that women with screen-detected tumours have lower mortality. This aligns with similar observations in the United States, where adjusted Cox models, using a 5-year follow-up period, demonstrated higher mortality among women with symptom-detected breast cancer compared to those with screen-detected breast cancer^31^. Previous research conducted on deprivation in Scotland found higher breast cancer mortality among the most deprived individuals, whereas among the T2DM cohort, no differences were observed^19^ potentially owing to this cohort undergoing continued surveillance for their diabetes condition reducing mortality not only from diabetes but for breast cancer as well.

Our results indicated that women who received surgery or hormone therapy had lower mortality when compared to those who did not receive the treatment, however, those who received chemotherapy or radiotherapy had higher mortality than those who did not receive the treatment. Breast cancer is often treated with a combination of therapies which depends on the molecular subtype. Women receiving hormone therapy and surgery are more likely to have early-stage cancer, a luminal subtype, or have better health^14,32^. Women with an aggressive subtype are more likely to have chemotherapy or radiotherapy treatments^15,32^. As previously mentioned, understanding the effect of type of treatment on mortality in observational studies is complex and requires further investigation. The use of the PREDICT breast cancer prognosis prediction tool would be highly beneficial in further analyses as it provides personalized prognostic information of 5-year, and 10-year survival estimates with and without treatments. Further research could validate the PREDICT tool for the T2DM population in larger datasets^33^.

While this study focused on prognostic factors for breast cancer specific survival in women with T2DM and breast cancer, it is crucial to acknowledge the competing risks of death from other causes. For example, individuals with T2DM face an elevated risk of cardiovascular disease (CVD), which has important effects on overall health and mortality and may act as a competing risk for breast cancer specific mortality^34^.

To our knowledge, this study is the first population-based investigation of prognostic factors for breast cancer specific survival in women with T2DM and breast cancer conducted in Scotland and the United Kingdom. A key strength of the investigation is the high-quality and nationwide data in SCI-diabetes with close to 100% completeness^16,17^. Moreover, these Scottish health datasets contain essential prognostic factors such as area-based deprivation (SIMD) and mode of detection that are not commonly available in many other nationwide databases.

Although this study was able to use high-quality data, the SMR06 registry posed a limitation due to incomplete molecular subtype information. The missingness is likely non-random, with women excluded from the cohort more likely to have poorer survival outcomes^35^. To address this, we conducted a sensitivity analysis using multiple imputation by chained equations, which highlights the importance of accounting for missing data. In our imputed models, HRs for all subtypes were attenuated, particularly for the most aggressive subtypes, such as TNBC and HER2-enriched. This suggests that the complete case analysis might have overestimated the relative risk for those subtypes. One possible explanation is that complete case analysis omitted a greater proportion of women with less aggressive subtypes, who were less likely to be treated and are generally older^36^. This potential selection bias by factors that affect breast-cancer specific mortality might have resulted in higher HRs in the complete case analysis due to the differences between the reference group (luminal A) which are generally healthier women and those with more aggressive subtypes^36^. Further, multiple imputation allowed for better adjustment of covariates and resulted in more precise estimates, which is particularly important in the case of small subgroups, such as TNBC and HER2-enriched subtypes.

As for any descriptive study, this study has the potential for bias and confounding. When investigating survival, these analyses are prone to lead time and length biases. Due to the breast cancer screening programme in Scotland, women had a better chance of survival than if no screening programme existed^37^. The average breast cancer screening uptake in Scotland from 2011 to 2021 was 72%^38^. Women with diabetes have been reported to have lower screening uptake than women without diabetes in some populations, which would be worth exploring further when linkage with breast screening data becomes available in Scotland^39,40^. Moreover, incorporating data on glycaemic control could help explore the potential effect on breast cancer outcomes^16^. Multivariable analyses were conducted to minimize the effects of confounding from known and available covariates. Although many important covariates such as treatments and deprivation were adjusted for, there were confounders not included that could have affected the results. For example, comorbidities, T2DM treatments, TNM stage, lifestyle factors (smoking status, physical activity, diet, alcohol consumption), and reproductive factors (age at menarche, number of births given, menopausal status) could be associated with subtype and survival. The 3-year follow-up, selected for comparison with the complete breast cancer population in Scotland^27^, is a limitation of the study. Longer follow-up could offer additional insights, and as follow-up data accumulate, it would be worthwhile to repeat the analysis.

In conclusion, this study highlights the effects of various prognostic factors on mortality in a cohort of women with T2DM and breast cancer. Prognostic factors such as molecular subtype and mode of detection of breast cancer mortality were identified, along with complex associations between treatments. The molecular subtype distribution and survival trends of women with T2DM and breast cancer were similar to the general breast cancer population in Scotland. However, our findings suggest limited potential differences between women in the general population and women with T2DM with breast cancer diagnosed 50–69 years of age, in rankings for breast cancer specific survival for TNBC and HER2-enriched subtypes. Given these differences and the complexity of treatment interactions in this population, there is scope for further investigation into breast cancer survival by subtype among women with T2DM in other larger populations including direct comparisons to survival among women without T2DM.

## Supplementary Information

Supplementary Information.

## Data Availability

The authors are not permitted to make the datasets generated and analysed during the current study publicly available. Data access can be requested from NHS Scotland’s Public Benefit and Privacy Panel for Health and Social Care. https://www.informationgovernance.scot.nhs.uk/pbpphsc/. For further information, please contact Dr. Sarah H Wild.

## Abbreviations

CHI: Community health index
CI: Confidence interval
ER: Oestrogen receptor
HER2: Human epidermal growth factor receptor 2
HR: Hazard ratio
ICD: International classification of diseases
IHC: Immunohistochemical
Ki-67: Ki-67 proliferation marker
KM: Kaplan Meier
NHS: National health service
Ref: Reference category
SCI-diabetes: Scottish care information diabetes
SDRN-NDS: Scottish diabetes research network national dataset
SES: Socioeconomic status
SIMD: Scottish index of multiple deprivation
SMR06: Scottish cancer registry
T2DM: Type 2 diabetes
TNBC: Triple negative breast cancer
