# Supplementary material for: Breast cancer survival and mortality among women with type 2 diabetes: a retrospective cohort study
Source: Sci Rep. 2025 Jul 18;15:26144. doi: 10.1038/s41598-025-08785-7 (PMC12274475; doi:10.1038/s41598-025-08785-7)
Supplement: Supplementary file 1 — Supplementary Information. [file 41598_2025_8785_MOESM1_ESM.docx]

**Supplementary file:**

**Table S1.** Three-year overall and breast cancer survival estimates (in %) (with 95% CI) by molecular subtype and age group at breast cancer diagnosis.

| Molecular Subtype | | Overall (all-cause) survival by age group at BC diagnosis | | | Breast cancer survival by age group at BC diagnosis | |
| --- | --- | --- | --- | --- | --- | --- |
|  |  | **50 - 69 years** | **70 - 84 years** | **50 - 69 years** | | **70 - 84 years** |
| Luminal A | no. risk / no. events | 746 / 55 | 587 / 190 | 746 / 27 | | 587 / 61 |
|  | three-year survival (95% CI) | 93.8 (92.2, 95.4) | 77.4 (74.6, 80.3) | 96.9 (95.8, 98.1) | | 92.0 (90.0, 93.9) |
| Luminal B (HER2-) | no. risk / no. events | 231 /28 | 180 / 83 | 231 /16 | | 180 / 36 |
|  | three-year survival (95% CI) | 90.1 (86.6, 93.6) | 71.0 (65.9, 76.5) | 94.3 (91.6, 97.0) | | 85.9 (81.6, 90.3) |
| Luminal B (HER2+) | no. risk / no. events | 107 / 19 | 69 / 38 | 107 / 14 | | 69 / 19 |
|  | three-year survival (95% CI) | 86.4 (80.9, 92.3) | 66.9 (58.8, 76.1) | 89.6 (84.5, 94.9) | | 81.6 (74.4, 89.5) |
| HER2-enriched | no. risk / no. events | 34 / 18 | 37 / 29 | 34 / 17 | | 37 / 17 |
|  | three-year survival (95% CI) | 70.4 (59.7, 83.0) | 59.1 (48.7, 71.8) | 72.0 (61.4, 84.4) | | 73.2 (63.0, 85.1) |
| TNBC | no. risk / no. events | 109 / 33 | 68 / 70 | 109 / 26 | | 68 / 53 |
|  | three-year survival (95% CI) | 78.2 (71.8, 85.1) | 52.2 (44.6, 61.0) | 82.5 (76.7, 88.9) | | 61.6 (53.9, 70.4) |

Abbreviations: BC = breast cancer, TNBC = triple negative breast cancer, HER2 = human epidermal growth factor 2, CI = confidence interval.
